# A Narrative Approach to Work With Groups of Bereaved Family Members

**DOI:** 10.1111/jmft.70094

**Published:** 2025-12-14

**Authors:** Silvia Renata Lordello, Isabela Machado da Silva

**Affiliations:** ^1^ University of Brasília; ^2^ Campus Universitário Darcy Ribeiro, Instituto Central de Ciências, Bloco A, Instituto de Psicologia Brasília DF Brazil

**Keywords:** bereavement, family systems theory, grief counseling, group intervention, narrative therapy

## Abstract

Families who have lost a member deal with an intense process of rebuilding their family dynamics and narratives. Losses during the COVID‐19 pandemic highlighted the lack of studies and intervention models for working with these families. In response to this, we developed an approach informed by narrative therapy practices. In this study, we aimed to understand the lived experience of 54 psychologists who facilitated support groups for bereaved family members. After each session, professional written reports were analyzed using reflexive thematic analysis. The findings highlighted the importance of a dialogical space to work with bereaved family members. Re‐membering and re‐authoring conversations were considered valuable approaches to work with families who have lost a member. This approach can inform clinical intervention supporting bereaved families in different contexts.

## 1 A Narrative Approach for Grief Counseling

Therapies informed by social constructionism represent one of the most important references for Brazilian family therapists (Pratti and Koller 2020). Narrative therapy is one of these guiding therapeutic approaches, applied across clinical settings, communities, workplaces, schools, the justice system, social assistance, and healthcare (Grandesso 2017, 2018, 2024a). In the face of public health emergencies such as the COVID‐19 pandemic, narrative therapy offers relevant approaches for mental health practice.

In this article, we present the development of a narrative‐therapy‐based group approach, designed to offer online support to those who have lost family members to COVID‐19. This article is based on the thematic analysis of the lived experience of 54 psychologists. They had all been trained to use this approach during a course offered through a partnership between the University of Brasília and the Federal District Health Department in Brazil.

To contextualize the development of this approach, we begin with a brief overview of family therapy in Brazil, especially regarding narrative therapy and social constructionism. Then, we discuss the impact of the COVID‐19 pandemic in Brazil and the theoretical model on which this approach is based.

### 1.1 Family Therapy in Brazil

Family therapy began to gain prominence in Brazil in the 1970s (Féres‐Carneiro and Ponciano 2005; Hintz and Souza 2009). At that time, family therapists trained in Argentina and the United States helped establish study and support groups, whose primary references were the systemic and psychoanalytic approaches.

Over time, Brazilian family therapists have integrated diverse epistemological, theoretical, and methodological points of view into their work (Paula‐Ravagnani et al. 2017; Pratti and Koller 2020). The approaches most used by Brazilian family therapists are the classic systemic models and postmodern approaches (Pratti and Koller 2020). When discussing postmodern approaches to family therapy, we refer to those informed by social constructionism (Paula‐Ravagnani et al. 2017), which will be discussed in the next section.

### 1.2 Social Constructionism and Narrative Therapy in Brazil

Social constructionism began to be explored in Brazil in the 1990s and gained prominence following the Portuguese translation of McNamee and Gergen's *Therapy as Social Construction* (Beiras et al. 2023; Focchi et al. 2023). According to the authors, the book emerged from debates that problematized traditional ideas about truth, objectivity, and knowledge, proposing that worldviews are socially constructed. This framework calls into question universal values, inviting us to be critically aware, curious about others, and hopeful in creating possible futures.

In 2024, the book *Practicing Therapy as Social Construction* (McNamee et al. 2024), with a foreword by Gergen, was published, taking stock of the 30 years since the 1990s volume. An important invitation was then presented: to look at microsocial practice connected to macrosocial discourse, enhancing its capacity to contribute to broader social change, based on dialogs about justice, race, power relations, and social inequalities.

According to Guanaes‐Lorenzi et al. (2014), although social constructionism was initially interpretated as a new form of therapy, this idea changed over time. McNamee and Gergen (2020) intended social constructionism to be understood as a philosophical or epistemological stance, a way of being in the world that invites dialog. It was never the intention of the constructionist movement to assert itself as a technique or to offer a set of intervention techniques. As a philosophical stance, premises such as flexibility, collaboration, values, and awareness about the role of history and social interactions in the process of constructing meanings are differentials that allow us to identify well‐marked changes between modern thinking and social constructionist thinking, which encourages practices that define themselves by their ethical‐political commitment, such as Narrative practices.

Narrative approaches challenge hegemonic discourses around problems, working to open new dialogs and to contest power relations, with social justice as a guiding horizon. To this end, people are encouraged to organize their experiences into stories that, in turn, will shape their lived experiences (Carrijo and Rasera 2010; Grandesso 2008; White and Epston 1990). The stories people tell about themselves, and their experiences can either limit or favor them, shaping the course of their relationships with others and with themselves. Throughout therapy, narrative therapists seek to create space for and encourage the development of alternative stories that dominant problem‐saturated accounts often obscure.

Narrative therapy has also been studied, taught, and practiced in Brazil since the 1990s (Grandesso 2024b). Its strong presence among family therapists in Brazil has provided space for creativity and favored the use of narrative therapy assumptions in diverse contexts (Grandesso 2017, 2018, 2024a).

One of these possible contexts is bereavement (White 2007). According to narrative therapy, it may be important for people to revisit the story of the relationship with their loved one who has died, reflecting on the role they played in each other's lives and what their relationship will be like from now on. Thus, death is not seen as a definitive end, but as an invitation to a renewed interaction; one that enables the loved one who has died to be present and give meaning to the person's life. This approach promotes a mourning process that strengthens identity and integrates meaningful relationships into the present life by bringing the memory of the loved one into a space of appreciation and internal dialog.

Accordingly, the bereaved establish a new meaningful place within their lives for the loved one who has died, acknowledging that person's significance for their own lives and identities. This work is grounded in considering how relational bonds mutually shape people, influencing how they understand themselves and their lives, and informing the decisions and choices they make.

(Silva et al. 2021, p. 04)

As it will be discussed in the following sections, this is a valuable approach in public health emergencies characterized by multiple, simultaneous, and complex losses.

### 1.3 The COVID‐19 Pandemic in Brazil

On January 30, 2020, the World Health Organization (WHO) recognized the spread of the new Coronavirus as a *Public Health Emergency of International Concern*, characterizing COVID‐19 as a pandemic 41 days later (World Health Organization n.d). In Brazil, the *Public Health Emergency of National Interest* was declared on February 3, 2020 (Brazilian Health Ministry 2020) and ended on May 22, 2022 (Brazilian Health Ministry 2022). By May 1, 2025, Brazil had accumulated 716,075 deaths related to COVID‐19 (Brazilian Health Ministry 2025), the second‐highest total number of deaths worldwide (World Health Organization 2025).

The possibility of being infected with the virus made contact with hospitalized loved ones difficult. Death often occurred suddenly, without the opportunity to say goodbye. Some people lost several family members in a short period of time. Funerals were restricted, making this farewell ritual more difficult. This whole context challenged the mourning processes and, consequently, the processing of these losses (Schmidt et al. 2022).

### 1.4 Developing a Narrative Group Approach to Support Brazilian Bereaved Family Members During the COVID‐19 Pandemic

By June 2020, more than 36,000 deaths caused by COVID‐19 had already been recorded in Brazil (Silva et al. 2021). The urgent need to develop intervention models to assist people who had lost loved ones and needed psychological support became evident.

Like other Brazilian federal and state universities maintained by the taxes paid by the Brazilian people, the University of Brasília aims to develop projects that benefit the community and expand the reach of the activities and knowledge built in the academic context. Several of these projects were proposed during the COVID‐19 pandemic, creating spaces for support and assistance to the population.

At that time, the Department of Clinical Psychology of the University of Brasília's professors were invited to develop and offer group therapy to people who had lost family members due to COVID‐19. With the support of volunteer psychologists, the two authors of this article responded to this invitation and developed an approach that used narrative therapy as its guiding principle. Michael White (1998, 2007) observed that, during bereavement, many people experience not only the pain of loss, but also a sense of rupture in their identity, given that identity is largely constructed through the bonds established over the course of life. Eventually, he proposed re*‐membering conversations* as a way of applying the *club of life* metaphor. White explained that when we lose a significant member of that club, we become vulnerable to losing important aspects of who we are. It is common to hear bereaved people say that “a part of me has gone too.” Thus, he proposed an innovative approach: to find ways bonds may live on even after death. The process of re‐membering invites people to keep the loved ones' stories, influences, and legacies actively integrated into their life narrative even after their death.

The revolutionary work of Hedtke and Winslade (2004) broadens this idea by bringing forward a way to implement this proposal. The authors developed inspiring material for bereavement group facilitators, encouraging practices that integrate the stories, values, and legacies of the deceased into the lives of the bereaved. Through narratives, rituals, and symbolic actions, participants are encouraged to maintain a meaningful relationship with their loved ones, recognizing their metaphorical presence in their lives.

In subsequent publications, the narrative approach to bereavement has been expanded and complexified, reinforcing a position contrary to conventional psychological theories of bereavement that often presume the dissolution of the relationship. From a narrative stance, ties between the living and the dead may remain enduring, accessible, and potentially transformative (Hedtke and Winslade 2017). This view of mourning has been disseminated in Latin America by Campillo (2021), who offered an articulation of re‐membering conversations inspired by these authors and explained how narrative practices, such as the tree of life and other approaches, may be significant therapeutic responses to the pain of loss.

The approach described here is based on reflexive questioning that focuses on two principal axes: (a) the ways in which the person who died remains alive within the identities of those who are still here, and (b) the marks that each bereaved person believes they have left on the lives of the deceased. Thus, the aim is to broaden the possibilities for re‐authoring the bereaved person's identity, enabling the deceased person's legacies to be acknowledged and honored, and to keep shaping the bereaved person's journey. Then, we propose a six‐session group format (see Table 1) in which the first two meetings establish a safe, welcoming context for group members to share their stories; the next two sessions focus on re‐membering conversations; and the final two meetings orient toward life‐project work.

**Table 1:** Session themes.

Session	Theme	Description
1	Getting to know the group and making agreements	Facilitators gain access to the group members' difficulties and skills by listening to the narratives spontaneously brought up by group members.
2	Investigating the support network and skills	Facilitators ask the group members what it is like to be part of a group that shares losses, but also many skills. They also invite the group members to reflect on who they may count on.
3	Introducing their loved one and their stories	Facilitators propose reflections on how family members who have passed away have contributed to the lives of the group members, and how this connection has influenced their identity and who they are today. They also invite group members to think about the ways they have influenced the lives of these family members.
4	Connection between the stories shared and the group members' resonances	Facilitators ask the group members how they felt touched by the stories shared in the group.
5	Searching for skills and community networks	Facilitators ask group members to recall the strategies and skills shared by colleagues throughout the sessions and ask which of these they might incorporate into their own lives.
6	Moving forward and revitalizing projects with the loved ones' legacies	Facilitators encourage the group members to reflect on how the people who have died will continue to be part of the clients' lives. They also propose reflections on the resumption of projects and the development of new ones.

The initial sessions aim to establish shared agreements for how the group will function and to invite the group members to share stories of the losses that brought them to the meeting. It is important to welcome these stories, as it is common for people to feel that they should not talk about it, either because they believe it will distress others or because others discourage such talk, due to the belief that remembering intensifies the pain (Silva et al. 2021). As Michael White (2007) notes, before creating space for and strengthening alternative stories, therapists first seek to understand the accounts that clients bring, as this will enable re‐authoring work. Re‐authoring conversations involves exploring people's alternative stories— stories often obscured by the dominant problem‐saturated stories—and constructing a new account of their lives out of neglected events that reveal significant intentions, values, and commitments (White and Epston 1990).

This creates a space for *double‐listening:* when group members talk about their losses, they also talk about the skills and knowledge that sustain them (Campillo 2009). As White (2006) mentioned, double listening is the practice of simultaneously listening to pain and to coexisting signs of resistance, hope, values, and intentions, even when they are not explicitly formulated. In Narrative Therapy, double listening is essential, since the therapist does not want to reduce the person to the problem‐saturated accounts of suffering. Accordingly, it is a way to receive stories of trauma, loss, or oppression, while also listening carefully to what the pain reveals about the person's commitments and intentions: to protect, to remain connected with treasured values, loved ones, and to sustain preferred hopes and dreams.

In the following meetings, facilitators seek to encourage both the process of re‐authoring and re‐membering (White 1998, 2007). They pose questions that invite the group members to think about the place the deceased loved one holds in their lives and how this dear one contributed to their identity. The group members are also invited to explore how they have contributed to the life and identity of the person who died. These inquiries expand possibilities for reflection and action on how the legacies left by these loved ones may be part of the group members' future.

The University of Brasília offered groups based on this approach on three occasions: July and October 2020, and February 2021. All three were conducted online, offered free of charge, and brought together people from across Brazil (Silva et al. 2021).

After the third edition, the University of Brasília and the Federal District Health Department joined in partnership to disseminate this approach among psychologists working in the Brazilian Unified Health System (SUS), through a theoretical‐practical course. The aim was to train these professionals to apply the approach in facilitating groups of family members bereaved by COVID‐19, integrating practice with theoretical and practical training.

### 1.5 Aims of the Study

This study aimed to understand the use of a psychosocial intervention approach, based on narrative therapy, in the context of online support for individuals who have lost family members due to COVID‐19. To this end, we investigated facilitators' accounts regarding (a) the group process and (b) the repercussions of the course on their professional careers.

## 2 Methods

### 2.1 Researchers' Positionality

Two psychologists with a PhD in psychology and a specialization in family therapy conducted this study. Social constructionism forms the epistemological basis for both and informs their practice as therapists and researchers. The first author of this study developed the approach presented in this article for facilitating groups of family members bereaved by COVID‐19 and worked as a facilitator and supervisor in the initial three groups offered by the university. She was a lecturer and supervisor on the course reported here. The second author was a supervisor in the initial three groups offered by the university but was not involved in the course.

### 2.2 Participants

Fifty‐four psychologists took part in a theoretical and practical course to act as group facilitators in an online group approach, based on narrative therapy, for people who lost family members due to COVID‐19. Thirty‐one of these professionals were the Federal District Health Department's employees, 42% of whom worked in primary health care, 42% in secondary care, and 16% in tertiary care. The other professionals who participated in the course worked in partner institutions, such as universities, colleges, and postgraduate courses. Table 2 shows the sociodemographic characteristics of this group of professionals.

**Table 2:** Sociodemographic description of the professionals who took the course.

Sex				
	Female	Male		
	85.2%	14.8%		
Age				
	From 21 to 44 years old		From 45 to 60 years old	Over 60
	87%		11%	2%
Race				
	White	Black		
	53.7%	44.5%		
Professional experience				
	Less than 5 years	From 5 to 15 years	More than 15 years	
	26%	56%	18%	
Education				
	Bachelor's Degree	Specialization	Master's Degree	Doctorate
	7%	74%	13%	6%

### 2.3 Design and Procedures

A case study (Stake, 1994) was carried out, focusing on the theoretical and practical training course developed through a partnership between the University of Brasília and the Federal District Health Department.

### 2.4 The Course

It lasted 60 h and was divided into synchronous and asynchronous activities. The synchronous activities included theoretical exposition, discussion of texts, and exchange of experiences (2 h per week), while the asynchronous activities included reading, preparation of materials, and planning of interventions (4 h per week). The theoretical content covered group therapeutic factors, social constructionism, narrative therapy, and the ethical precautions to be adopted in online support group. An innovative aspect of the course was that it also offered the community therapeutic support groups, so the session‐by‐session follow‐up was part of the training itself.

The course took place between May and August 2021. After completing the theoretical modules, the groups were formed and led by three facilitators (two professionals and a trainee from the Psychology course at the University of Brasília), who received ongoing support via a virtual forum. The composition of the groups was based on the professionals' schedules. Trainees accompanied the groups to support the professionals, observe the sessions, and make records. It was also felt that this would be an important opportunity for these students to develop relevant skills for their future professional practice.

The groups were publicized on social networks, and those interested in participating signed up using online forms. In total, 255 people from different regions of Brazil were divided into groups with up to 15 members. Table 3 presents the group members.

**Table 3:** Characterization of the group members.

Gender								
Cis woman	Trans woman	Cis man	Trans man	Nonbinary				
90%	0.5%	9%	0%	0.5%				
Age								
16 a 21	22 a 44	45 a 60	Over 60	Average: 42.3 (SD: 12.3)				
5%	57%	30%	8%	Range: 16–83				
Race								
White	Black	Asian	Indigenous	Not Identified				
39%	56%	2%	0.4%	0.6%				
Marital status								
Married	Divorced	Widowed	Single					
38%	7%	22%	33%					
Education								
Incomplete Elementary School		Complete Elementary School	Incomplete Secondary School	Complete High School	Incomplete Degree	Full Degree	Postgraduate	
3.5%		4%	3.5%	15%	12%	28%	34%	
Family members lost in the pandemic								
Child	Parent	Sibling	Husband, wife, or fiancè	Grandparents	Uncle, Aunt, Cousin, nieces, or nephews	In‐laws	Friend	Multiple losses
4%	35%	10%	19%	1%	5%	2%	2%	22%

### 2.5 Access to the Facilitators' Lived Experience

Facilitators' lived experience were accessed through their reports in the forums within a virtual learning environment (Moodle). Forums are important tools in distance education processes and provide space where students can ask questions, solve doubts and share ideas, experiences, and reflections (Pires & Veloso, 2023). Each forum addressed a specific topic: (1) group therapeutic factors, the foundations of social constructionism, and the use of narrative therapy in group work; (2) the relational and psychological effects of the COVID‐19 pandemic^1^, and (3) group planning. From the fourth to the ninth forum, facilitators were invited to describe the sessions they had led immediately after each meeting. Their accounts were also accessed through a metacognitive report in which facilitators evaluated their progress in the course.

### 2.6 Data Analysis

We analyzed the facilitators' session reports to understand their accounts of the group's process. We also analyzed the forums in which they recounted their studies, the group planning, and their report on their experience in the course.

We carried out a reflexive thematic analysis with both accounts (Braun and Clarke 2021). This method allows greater depth in capturing meanings and gives room for the researchers' interpretation in generating themes from the facilitator's descriptions. The researchers conducted the analyses independently and then discussed them with each other. It should be noted that in thematic analysis, when two or more researchers analyze the same material, they do not intend to agree with each other (Terry et al. 2017). Having multiple researchers analyze the data broadens the possibilities of interpretation. For the same purpose, we also used the software *Requalify. ai* (Version 0.1; Martins et al. 2024), which aids the coding process. The software was used after researchers' coding, and its results complemented the descriptions and discussions of the themes.

The analysis followed eight stages: (a) repeated and careful reading of the material by both researchers, independently, to familiarize themselves with the accounts presented by the participants; (b) elaboration of the initial codes by the researchers; (c) analysis of the material using the *Requalify.ai* software; (d) comparison between the codes elaborated by the researchers and the software analysis; (e) meetings between both researchers to develop the codes; (f) development of themes by both researchers, independently; (g) meetings between both researchers to review and improve the themes; and (h) writing the text.

### 2.7 Quality Criteria

We tried to adopt quality criteria that aligned with our epistemological basis and recognized the importance of the subjectivity of all those involved (Morrow 2005). Both researchers analyzed and discussed the data to integrate and expand the attributed meanings. The researchers had different relationships with the course reported here, which allowed them both to analyze the data from their particular point of view: one had a close relationship with the course, acting as an instructor and supervisor; the other did not take part in the course, offering a fresh look at the data she was accessing for the first time. The researchers also tried to be fair to the course participants' accounts, using their own words whenever possible to better represent the meanings they attributed to the experience. Similarly, contradictory versions were included to express the facilitators' different views.

### 2.8 Ethical Considerations

This study followed the ethical principles set forth in Resolution 510 of April 7, 2016, which governs research in the humanities and social sciences in Brazil (Brazilian National Council of Justice 2016). These principles include respect for the participants' autonomy, freedom, diversity, and values. Their confidentiality and anonymity were duly preserved. According to this resolution, studies characterized by the analysis of data that arise spontaneously from the regular exercise of professional practice, without conducting specific procedures aimed at answering a particular research question, are not required to be submitted to a research ethics committee. This study describes a course aimed at training professionals to facilitate groups for family members bereaved by COVID‐19. When the course was designed, the aim was not to conduct research; the focus was on training professionals and serving the population. After the course concluded, we chose to document the experience by analyzing material posted in the course forums. All data used in this study were presented spontaneously by the participants in the forums during the course. No interviews were conducted, and no specific questionnaires were submitted for research purposes. The results and discussion presented here refer to analyses and reflections made after the conclusion of a professional intervention. In accordance with Brazilian regulations, provided that participants are not identifiable, a precaution adopted in this study, submission to an ethics committee was not required.

## 3 Results and Discussion

### 3.1 The Group's Process

The group process was analyzed by developing two themes. Table 4 presents these themes along with their subthemes and examples of the codes.

**Table 4:** The group process: themes, subthemes, and codes.

Themes	Subthemes	Code examples
Creating a safe space for the heart to grieve	Welcoming and respecting the particularities of each journey	Complexities and particularities of bereavement processes The need for a space that welcomes and respects the various bereavement processes
	The collectivity favoring the rebuilding processes	Active role of group members The importance of speaking and listening for group members' bonding process Validation and acceptance among group members The meetings provided a space for deep reflection on the pain of loss and the possibility of rebuilding Creation of new emotional support networks Integration of group members into each other's personal stories
The continuous interrelationship of past, present, and future in the resignification of personal lived experiences	Re‐membering as the resignification of absences	Re‐authoring memories The contribution of the family members who have died to the lives of the group members Valuing emotional memories The transformative potential of loss Keeping the presence of the deceased relatives alive
	Continuing life: reconnecting with projects beyond mourning	Celebrating the lives and legacies of the family members who have died Re‐engage with everyday life Living one day at a time

#### 3.1.1 Creating a Safe Space for the Heart to Grieve

The first theme refers to the process experienced by both the facilitators and the group members co‐creating a space of mutual support. In this theme, the dialectical relation between the particular and the collective is present: participants highlighted the importance of a context where particularities and differences are welcomed and respected, as well as the potential for collective creation and transformation.

##### 3.1.1.1 Welcoming and Respecting the Particularities of Each Journey

Throughout the sessions, the group members' storylines interwove shared experiences that bound them together with accounts that emphasized the uniqueness of each person's grieving process.

At times, the plurality of these stories makes people reflexive and quite emotional because there are people who see their loved ones as great heroes. At the same time, others feel very angry with the deceased for not having taken enough care regarding social distancing.

(Facilitator 25)

Recognizing and respecting the uniqueness of each journey is central to narrative approaches. Michael White (2009) emphasized the singularity of each storyline and therapeutic journey, since they unfold “in ways that could not have been predicted ahead of the journey itself and in the sense that each journey brings with it options for entering new territories of thought and practice” (p. 19).

Another aspect that demonstrated the particularities of the stories shared by group members was the way they related with their support network. Some acknowledged the relevance of their network, others felt they could not talk about their losses with those close to them: “We also had reports of loneliness sometime after the death, due to the pressure to get on with life. One participant reported this contributed to distancing her from a support network” (Facilitator 52). On the other hand, some felt they “couldn't let anyone get close because of the pain and because people didn't understand the size of the pain” (Facilitator 3), as well as those who preferred to experience this moment by themselves:

One of the group members, whose husband died of COVID‐19, told us that her friends and family were always trying to help, but, at the moment, she would rather be “in silence”, despite recognizing the care behind their efforts. According to her, “my grief is lonely”. At this point, the group responded with dedicated support; two participants welcomed her speech and emphasized the uniqueness of this experience of bereavement as something singular and not comparable across people.

(Facilitator 36)

These meetings made evident the importance of creating a space where people can talk about their losses as much as needed (Campillo 2009; White 1998, 2007). The group members' grieving processes proved to be unique and nonlinear, inter‐weaving moments that emphasized the loss with moments that highlighted the skills and knowledge that sustain them. During the process, each person experienced different points in their processes. Although facilitators were attentive to this plurality, it is worth noting that the group itself also demonstrated its skills to accommodate it:

As the group members begin to share unique affections, sensations, and emotions accounts, we can notice the group's movements to establishing its own dynamics. This invites us, as therapists, to redefine our role during the entire process. For me, this is one of the richest aspects of being a therapist in a therapeutic group.

(Facilitator 10)

Given the singularity of people's stories and therapeutic journeys, this group‐based approach may not be suitable for everyone bereaved by the death of a family member. Facilitators reported that some group members discontinued attendance, stating they did not yet feel ready to speak about their loss. Others paused for several sessions and returned later, suggesting they needed more time before re‐engaging with the group.

##### 3.1.1.2 The Collectivity Favoring the Rebuilding Processes

The bonds developed among group members enabled them to revisit their pain, joy, and life stories from a supportive and welcoming stance. The complex feelings carried out since the deaths of family members, as well as the shared challenges to the losses experienced during the pandemic, emerged as elements of connection among the group members, fostering identification, empathy, and solidarity, as mentioned by Facilitator 21:

The group members referred to each other as colleagues or companions, identified similarities in their feelings, and welcomed each other. Some quotes from the group members really touched me, such as: “I thank my colleague, because she has put into words exactly what I feel yet couldn't express: the feeling that I no longer exist”.

Facilitators also emphasized the importance of both speaking and listening. As one person tells his/her story, others listen and offer validation, while the person hears his/her own words from a repositioned stance. Stories become interwoven; with each person's contribution, exchange, and shared recognition the collective narrative is thickened. In witnessing the pain of someone experiencing a situation so close to their own, the group members both demonstrated and recognized some of their own personal agency.

Therapeutic approaches based on social constructionism emphasize the importance of combining knowledge and perspectives to expand the possibilities for action (Andersen 2002; Carrijo and Rasera 2010; Grandesso 2008; Paula‐Ravagnani et al. 2017). We understand the group's polyvocality and the bonds among its members enhanced this process. The practice of double listening (Campillo 2009), that is, paying attention simultaneously to the problem‐story and to alternative accounts, acknowledging the wound while creating space for new stories to emerge, fostered re‐authoring and was sustained not only by the facilitators but, above all, by fellow group members. More than expressing support, the group members practiced double listening, looking beyond hopelessness to acknowledge, name and honor their loved one's intense struggle and efforts to uphold dignity.

Facilitators reported that the group members increasingly became part of one another's lives, and the topics addressed in sessions began to shift. They exchanged suggestions on practical everyday issues, such as strategies for increasing income, further strengthening bonds, and demonstrating a growing sense of belonging and trust. By the end of the sessions, members of some groups had established ways of staying connected with each other, such as sharing phone numbers and creating WhatsApp groups. These bereavement groups seem to have expanded the group members' “clubs of life,” thereby enriching their narratives and broadening their possibilities for action (White 2009). In this sense, bereavement groups such as the one described here may perform functions similar to the *definitional ceremonies* proposed by White, once their members act as *outsider witnesses* for one another, extending the boundaries of the initially presented storylines.

#### 3.1.2 The Continuous Interrelationship of Past, Present, and Future in the Resignification of Personal Lived Experiences

This theme addresses the facilitators' accounts on the use of narrative practices in processes of re‐authoring and expanding personal narratives. The two subthemes that will be presented illustrate the intricate and inherent relationships of past, present, and future. They show that lived experiences influence one another in multiple directions in the ongoing construction of meaning (Carrijo and Rasera 2010; Grandesso 2008; White and Epston 1990). While the past contributes to the meanings attributed to the present and to what is considered possible for the future, the present, in turn, gives new meaning to the past and opens new possibilities for action.

##### 3.1.2.1 Re‐Membering as the Resignification of Absences

This subtheme indicates that the questions posed by the facilitators (“What was the deceased's contribution to your life?” and “What was your contribution to that person's life?”) were understood as “fundamental to the construction of valued narratives.” The group members introduced their loved ones in multiple ways, through videos, photos, poems, songs, and stories, by naming characteristics, and recalling life episodes that illustrated the relationship and the bonds between the bereaved and the deceased family member. From the facilitators' point of view, these were emotional moments that enabled participants to make new meaning of their experiences and engage in deep reflection:

One group member reflected that the only way her children would come to know their father would be through the stories she would tell. So, she decided to create a place at home that would tell the story of their father's life, honoring his life, not his death. She also affirmed that they would now be responsible for keeping their relatives alive for those who remain. […] A beautiful statement: “Now his heart beats in mine”; “His departure does not undo his existence”.

(Facilitator 5)

A young participant who had lost her mother and had kept silent for a long time was moved to recognize that her mother's legacy is within her. “I had never thought about it this way. This has given me strength,” she said.

(Facilitator 33)

The proposed activity achieved its aim of promoting re‐membering conversations. This narrative practice seeks to strengthen and reconstitute the relationships between the persons and those who have been significant and have contributed to shaping their identities, whether these significant people are alive or not (Campillo 2009; White 1998, 2007). The concept is based on the understanding that meaningful relationships do not necessarily end with physical absence but may continue to influence and enrich a person's life symbolically and emotionally. There was also an emphasis on listening to and telling stories about the deceased, a practice regarded within narrative therapy as important to the grieving process. The power of storylines was evident in the accounts that presented them as means of transforming the pain of loss into sustaining meanings, reaffirming the person's relevance in their life and identifying how these ties continue to contribute to their identity and emotional well‐being. In the groups' therapeutic journeys, re‐membering conversations opened a new phase in which stories shared by the group members favored processes of resignification and the construction of alternative stories, as stated by Facilitator 42:

After the initial phase of the group, which was essentially based on problem‐saturated versions, it was very gratifying to see how re‐membering conversations enabled the group to move on to a new stage, marked by the resignification of absences. Re‐membering opened space for re‐authoring conversations; each participant was able to move from mournful to preferred storylines, naming how the presence of the loved one endures in their daily lives, thereby reauthoring the loss and the experience of mourning.

Facilitators supported these outcomes were inviting the group members to explore stories, memories and shared experiences with their loved ones; a line of inquiry central to sustaining the deceased as members of one's “club of life” (White 1998, 2007). According to White (1998, 2007), questions oriented towards shared contributions, values, and skills function as scaffolding for thickening preferred identities and promoting personal agency.

##### 3.1.2.2 Continuing Life: Reconnecting With Projects Beyond Mourning

This subtheme addresses facilitators' point of view on how group members integrated the experience of loss and the memories of their deceased relatives into their projects for the future. According to participants' accounts, they felt free to share their perceptions, which ranged from diminished interest and a sense of not yet being ready to resume projects, to recognizing that re‐engagement often begins with small steps: “1 day at a time,” “little by little, I'm standing still” (Facilitator 28).

In general, the projects were being resumed. They were related to everyday issues and the resumption of activities in the present, which I consider essential movements that scaffold more elaborate projects. Being with family, celebrating important moments again, returning to work, and re‐engaging with social life were examples of resumed projects. I have heard them saying they would not be able to cope but, as time went by, they felt they were gradually returning to their life projects. Some reported that they could not plan their lives for the distant future, but felt satisfied returning to their daily lives, one day at a time.

(Facilitator 23)

The role this type of group can play in situations as adverse as those experienced by people who have lost their family members in a pandemic was evident: “They reported feeling comforted by the unprecedented exercise of giving new meaning to the presence of their loved one, re‐membering life and its legacy for themselves and the world.” (Facilitator 51).

From the facilitators' vantage point, these were emotionally significant moments that opened space for reflection and re‐authoring. Participants praised narrative practices such as re‐authoring and double listening and intensely adopted them throughout all the sessions. These results are consistent with the contributions of Michael White (1998) and Marta Campillo (2009), who invited us to redefining mourning as an ongoing relationship and a meaningful resignification process which challenges hegemonic practices that promote detachment. By bringing the memory of the loved one into appreciative, relational dialog, the approach strengthened preferred identity conclusions and integrated meaningful relationships into present life and future projects of living.

### 3.2 The Facilitators' Process

Two themes, shown in Table 5, helped us understand the facilitators' process and the meanings attributed to this experience.

**Table 5:** Facilitators' process: themes, subthemes, and codes.

Themes	Subthemes	Code examples
Participation in the course as an opportunity to re‐author professional experiences	Social constructionism and narrative therapy promoting reflection and dialog	Personal and professional development and transformationAcknowledging professional growth
	The relationship with the other broadening professional experiences and practices	Valuing supervision and collective learning Mutual support among professionals Active listening
Rebuilding boundaries in the face of the new	Proximity between the personal and the professional in a health emergency context	Fears about how to deal with resonances Similarities in the experiences of professionals and group members
	Blurring boundaries between the university and the public health system	The importance of the partnership between the university and the SUS Institutional support and collaboration networks Desire to continue to work with the groups Proposals for new actions Importance of continuing education

#### 3.2.1 Participation in the Course as an Opportunity to Re‐Author Professional Experiences

This theme examines the facilitators' complex trajectories throughout the course and how participation supported the reauthoring of their professional accounts. Facilitators highlighted the impact of their engagement with social constructionism and narrative therapy, and the importance of creating a dialogical space characterized by collective learning, as discussed in the following subthemes.

##### 3.2.1.1 Social Constructionism and Narrative Therapy Promoting Reflection and Dialog

This subtheme comprises the professionals' reflections on the position they came to occupy after engaging with texts and discussions on narrative therapy and social constructionism. They revisited and reconstructed their accounts of what it means to be human, the nature of therapeutic conversations, and what it means to participate in them, regardless of each one's role. Social constructionism is not just a set of guidelines for how the problems presented by those seeking professional support should be understood and addressed (Guanaes‐Lorenzi et al. 2014; McNamee and Gergen 2020). It is a worldview encompassing ontological and epistemological commitments that shape how we act in the world. Facilitators highlighted the importance of understanding human beings as a social beings, constituted in language and in the meanings they attribute to their interactions:

I believe postmodern approaches are highly relevant when considering a therapeutic group. In my view, these approaches bring an innovative understanding that is essential in this setting: the social lens. From this vantage point, the issues raised are not the product of individuals in isolation but are socially constructed across different contexts and realities. In my opinion, having this notion combined with practice is fundamental when conducting a therapeutic group.

(Facilitator 48)

Facilitators also highlighted polyvocality and the need to be open to diversity:

For me, the assumptions of constructionism make a lot of sense when it comes to experiencing a clinic that enables the expression of multiple ways of being in the world. The rejection of absolute truth, for example, opens us to truths produced by individuals and collectives, creating opportunities for listening that does not seek an *a priori* outcome but co‐creates possibilities collectively. I believe that the assumptions of postmodern therapies allow us to expand our practices in the SUS toward more inclusive, autonomy‐enhancing approaches.

(Facilitator 33)

Therefore, facilitators' accounts recognized that social constructionism and narrative therapy are aligned with specific values in therapeutic practice. As Manfrim and Rasera (2016) pointed out, the therapist informed by social constructionism has an ethical commitment to social change, as highlighted by Facilitator 15.

The paper listed in the references recalls the psychologist's code of ethics and our ethical and political commitments. Accordingly, a psychology that critically questions inequalities, prejudice, and other problems that affect lived existence is essential.

In this sense, the assumptions of narrative therapy call on facilitators to see those with whom they work broadly, recognizing their complexities and their potential:

Reading texts with dense and new concepts… An important point in my professional practice is never forgetting to focus on potential. Often, given everyday demands, it is possible to get caught up and narrow listening to damage, faults, and problems. Learning about narrative therapy and realizing that it involves understanding people's life stories and re‐authoring them, has helped me look more closely at clients with the understanding that there is more than just the problem.

(Facilitator 53)

To this end, the importance of assuming a *not‐knowing position* (Anderson and Goolishian 2020) was reiterated, establishing a nonhierarchical relationship:

Based on my reading of the materials provided, I see group work informed by postmodern approaches as reflective work that can foster a space for exchange, where the group members may bring their experiences without judgments and consider singular aspects, such as their beliefs, culture, language, and practices, as important. A space in which the professional is a mediator who provides space for the other to speak, without imposing norms or codified knowledge.

(Facilitator 44)

The studies conducted in the initial phase of the course also showed the potential to assign new meanings to the practices that facilitators were already undertaking in their work:

The text on postmodern approaches articulated and reinforced much of what is developed in my service. […] leading me to conclude that much of what I do, together with the team I am part of, has broader scope and greater coherence than I had assumed.

(Facilitator 22)

##### 3.2.1.2 The Relationship With the Other Expanding Narratives and Professional Practices

This subtheme draws on participants' perceptions about how the relationships established throughout the course contributed to their professional accounts. Planning an online group to address a topic as sensitive as the death of a family member elicited complex emotions; participants reported fear, enthusiasm, and gratification.

I confess that right from the beginning, I found myself anxious, excited, and worried about the challenges this work might bring. However, as our meetings progressed, through alignment with my partner and trainee, and through the readings, anxiety and worry gave way to excitement and anticipation for an activity that will probably be transformative for both the group members and us as therapists.

(Facilitator 43)

As emphasized by social constructionism, the importance of horizontal spaces characterized by respect for differences and the co‐construction of meanings was evident in the creation of a safe space for the facilitators to learn and broaden their view of themselves as professionals:

I believe that leading a group as a team will be a protective and stabilizing factor for me and for the group, which will be able to draw on a plurality of experiences and knowledge. One person I contacted also mentioned this value, saying that she “signed up precisely to listen to other people.”

(Facilitator 25)

#### 3.2.2 Rebuilding Boundaries in the Face of the New

The COVID‐19 pandemic has prompted societies to review priorities, values, and modes of functioning, including interpersonal relationships and interorganizational dynamics. This theme addresses the blurring of boundaries between the personal and the professional, as well as between universities and other public services, which favors new forms of organization in response to these challenges.

##### 3.2.2.1 Proximity Between the Personal and the Professional in a Health Emergency Context

This subtheme brings together professionals' accounts of their experiences during the pandemic. They reported impacts on their own lives, including the loss of loved ones, which helped the facilitators feel closer to the group members. While these individual experiences helped them develop a more humane, empathic listening stance, they also described being transformed by the opportunity to witness the group members' accounts.

Through these memories and stories, I later reconnected with loved ones who had died, but in different circumstances. I resonated with aspects of their feelings and stories. This experience showed me the importance of the internalized presence of loved ones, the imprint of affection and love. It also highlighted the importance of memory and the possibility of missing them, recognizing that they remain present in our lives as members of our “club of life”. […] I believe that my life experience and how I have dealt with loss, together with my theoretical commitments, contributed to refining my listening and welcoming stance, as well as to formulating more sensitive questions in sessions.

(Facilitator 54)

##### 3.2.2.2 Blurring Boundaries Between the University and Other Public Services

This subtheme highlights the value of institutional partnerships and the importance of interinstitutional coordination. In their accounts, facilitators emphasized the partnership between the university and the health care department, which enabled a course with an innovative approach that integrated theory with supervised practice, introduced less widely known content, such as social constructionism and narrative therapy. This collaboration was viewed as essential to professional training and to participants' continuing education.

The development and recognition of skills throughout this experience contributed to the idea of offering similar groups in diverse settings, such as prisons, hospitals, primary health care units, and services for those whose loved ones have been victims of violence against women. Plans were also made to disseminate the learning from this course to other health professionals, such as physicians and nurses. Even those apprehensive about their capacity to continue this work in the future articulated thicker accounts of what might now be possible.

This is one of my fears about my capacity to disseminate this knowledge to other professionals, as I am still learning this rich and special approach. […] Regarding skills, maintaining a non‐prescriptive stance is something I will continue to develop. With this experience, I strengthened my empathic stance and shared what resonated with me. I felt more at ease to feel and to show what I felt, without fear of seeming less professional or less qualified to be there. Feeling permitted to make mistakes was also great!

(Facilitator 16)

As Michael White (2009) noted, psychotherapists' accounts are often impoverished by dominant professional and institutional discourses that delimit what counts as legitimate knowledge and constrain possibilities for professional action. Meetings that allow these accounts to be thickened, through exchanges that enable re‐authoring, tend to support professionals' mental health.

## 4 Conclusion

The considerable number of deaths related to COVID‐19 across countries, the prospect of future public health emergencies, and the increasing frequency of climate disasters reinforce the need to train mental health professionals to deal with multiple, simultaneous, and complex losses. The group‐based narrative approach presented in this study was designed to provide professionals with tools for responding to paralyzing moments, such as those experienced during the pandemic, while reaffirming an ethical‐political commitment to transforming adverse realities. However, its applicability goes further: according to facilitators' accounts, the proposed clinical interventions, with demonstrated therapeutic potential, suggest that learning derived from group processes can be applied to various coping situations.

Facilitators indicated that the proposed approach represented a distinctive contribution to their training. Continuing education integrating supervised clinical practices, narrative practices aligned with a dialogical orientation, and collaborative learning were highlighted as pillars of the groups' success. Participants mentioned the quality of the support materials and evaluated real‐time supervision and collaborative forums positively. The online narrative approach for group work also proved highly cost‐effective, enabling concurrent support for multiple people and overcoming geographical barriers and travel limitations, without affecting the depth of the group experience.

The partnership between the public university, which developed the narrative approach for group work, and public health system, as a social policy, was central to the project's success. This collaboration showed how academic production can arise from concrete social demands and be returned to them with transformative effects. Facilitators reported the importance of an approach that promotes emancipation, dignity, and the protection of rights, in alignment with the principles of narrative therapy and its ethical commitment to a more just and humane world.

Finally, the study's primary limitation was the lack of a systematic analysis of the group members' accounts. Such analysis could have enabled a more polyphonic and complex representation of the experience, further enriching the findings and reflections presented here.

## Endnotes

1 The narratives presented in the second forum were not analyzed in this study.

## References

[jmft70094-bib-0001] Andersen, T. 2002. Processos Reflexivos (2nd ed. Instituto Noos).

[jmft70094-bib-0002] Anderson, H. , and H. Goolishian . 2020. “O cliente é o Especialista: A Abordagem Terapêutica do não Saber.” In A Terapia Como Construção Social, edited by S. McNamee and K. J. Gergen , (2nd ed., 53–72. Instituto Noos.

[jmft70094-bib-0003] Beiras, A. , S. McNamee , E. F. Rasera , and P. Martins . 2023. “Conhecendo O Livro “Praticando a Terapia Como Construção Social.” Nova Perspectiva Sistêmica 32, no. 75: 117–121.

[jmft70094-bib-0004] Braun, V. , and V. Clarke . 2021. Thematic Analysis: A Practical Guide. Sage Publications.

[jmft70094-bib-0005] Brazilian Health Ministry . 2020. Portaria n° 188, de 3 de Fevereiro de 2020. Declara Emergência em Saúde Pública de Importância Nacional (ESPIN) em Decorrência da Infecção Humana Pelo Novo Coronavírus (2019‐nCoV). *Diário Oficial da União*. https://www.in.gov.br/en/web/dou/-/portaria-n-188-de-3-de-fevereiro-de-2020-241408388.

[jmft70094-bib-0006] Brazilian Health Ministry . 2022. Após Dois Anos, Chega ao fim Estado de Emergência em Saúde Pública Por Conta da Covid‐19 no Brasil. *Ministério da Saúde: Notícias*. https://www.gov.br/saude/pt-br/assuntos/noticias/2022/maio/apos-dois-anos-chega-ao-fim-estado-de-emergencia-em-saude-publica-por-conta-da-covid-19-no-brasil.

[jmft70094-bib-0007] Brazilian Health Ministry . 2025. Painel Coronavírus. Coronavírus Brasil. https://covid.saude.gov.br/.

[jmft70094-bib-0008] Brazilian National Council of Justice . 2016. Resolução n° 510, de 07 de abril de 2016. Brazilian Health Ministry. https://www.gov.br/conselho-nacional-de-saude/pt-br/atos-normativos/resolucoes/2016/resolucao-no-510.pdf/view.

[jmft70094-bib-0009] Campillo, M. 2009. Terapia Narrativa: Autoaprendizage y Co‐aprendizage Grupal. Ediciones Ollin.

[jmft70094-bib-0010] Campillo, M. 2021. “Terapia Narrativa: Respondiendo Al Duelo Y La Perdida Con El Árbol De La Re‐Asociación.” Revista de Psicoterapia 32, no. 119: 181–195. 10.33898/rdp.v32i119.422.

[jmft70094-bib-0011] Carrijo, R. S. , and E. F. Rasera . 2010. “Mudança Em Psicoterapia De Grupo: Reflexões a Partir Da Terapia Narrativa.” Psicologia Clínica 22, no. 1: 125–140. https://www.redalyc.org/pdf/2910/291022021008.pdf.

[jmft70094-bib-0012] Féres‐Carneiro, T. , and E. Ponciano . 2005. “Articulando Diferentes Enfoques Teóricos Na Terapia Familiar.” Revista Interamericana de Psicologia 39, no. 3: 439–448. https://www.redalyc.org/pdf/284/28439314.pdf.

[jmft70094-bib-0013] Focchi, L. A. , E. F. Rasera , and C. Guanaes‐Lorenzi . 2023. “Aproximações Recentes Entre Construcionismo Social E Terapia No Contexto Acadêmico Brasileiro.” Pensando Famílias 27, no. 1: 3–23. https://pensandofamilias.domusterapia.com.br/index.php/files/article/view/80/32.

[jmft70094-bib-0014] Grandesso, M. 2008. Sobre a Reconstrução do Significado: Uma Análise Epistemológica e Hermenêutica da Prática Clínica. Casa do Psicólogo.

[jmft70094-bib-0015] Grandesso, M. 2017. Práticas Colaborativas e dialógicas em Distintos Contextos e Populações: Um Diálogo Entre Teoria e Práticas. CRV.

[jmft70094-bib-0016] Grandesso, M. 2018. Colaboração e Diálogo: Aportes Teóricos e Possibilidades Práticas. CRV.

[jmft70094-bib-0017] Grandesso, M. 2024a. A Construção Social de Práticas Relacionais: Diálogo e Colaboração em ação. Martins Fontes‐Paulista.

[jmft70094-bib-0018] Grandesso, M. 2024b. “Terapia Narrative No Brasil.” Journal of Contemporary Narrative Therapy 1: 2–13. https://www.journalcnt.com/uploads/9/4/4/5/94454805/february_2024_grandesso_portuguese.pdf.

[jmft70094-bib-0019] Guanaes‐Lorenzi, C. G. , M. dos S. Moscheta , C. M. Webster , and L. V. Souza . 2014. Construcionismo Social: Discurso, Prática e Produção do Conhecimento. Instituto Noos.

[jmft70094-bib-0020] Hedtke, L. , and J. Winslade . 2004. Remembering Lives: Conversations With the Dying and the Bereaved. Routledge.

[jmft70094-bib-0021] Hedtke, L. , and J. Winslade . 2017. The Crafting of Grief: Constructing Aesthetic Responses to Loss. Routledge/Taylor & Francis Group.

[jmft70094-bib-0022] Hintz, H. C. , and M. O. Souza . 2009. “A Terapia Familiar no Brasil.” In Manual de Terapia Familiar, edited by L. C. Osorio and M. E. P. do Valle , 91–103. Artmed.

[jmft70094-bib-0023] Lordello, S. R. , and I. M. Silva . 2021. “The Grief Elaboration Process in the Pandemic Scenario: A Group Intervention.” In Anxiety, Uncertainty, and Resilience During the Pandemic Period ‐ Anthropological and Psychological Perspectives, edited by F. Gabrielli and F. Irtelli , 215–225. IntechOpen. 10.5772/intechopen.98837.

[jmft70094-bib-0024] Manfrim, A. F. N. , and E. F. Rasera . 2016. “Diálogos Entre O Discurso Construcionista Social E a Terapia Social.” Nova Perspectiva Sistêmica 56: 34–48. https://pepsic.bvsalud.org/scielo.php?script=sci_arttext&pid=S0104-78412016000300004.

[jmft70094-bib-0025] Martins, L. F. , F. R. Souza , and J. M. Freitas . 2024. *requalify.ai* (Version 0.1) [online software]. https://requalify.ai.

[jmft70094-bib-0026] McNamee, S. , and H. J. Gergen . 2020. “Introdução à Segunda Edição Brasileira.” In A Terapia Como construção Social, edited by S. McNamee and K. J. Gergen , (2nd ed., 11–17. Instituto Noos.

[jmft70094-bib-0027] McNamee, S. , E. Rasera , and P. Martins . 2024. Praticando a Terapia Como Construção Social. Noos.

[jmft70094-bib-0028] Morrow, S. L. 2005. “Quality and Trustworthiness in Qualitative Research in Counseling Psychology.” Journal of Counseling Psychology 52, no. 2: 250–260. 10.1037/0022-0167.52.2.250.

[jmft70094-bib-0029] Paula‐Ravagnani, G. S. , C. Guanaes‐Lorenzi , and E. F. Rasera . 2017. “Use of Theoretical Models in Family Therapy: Focus on Social Constructionism.” Paidéia (Ribeirão Preto) 27, no. 67: 84–92. 10.1590/1982-43272767201710.

[jmft70094-bib-0030] Pratti, L. E. , and S. H. Koller . 2020. “Práticas da Terapia de Família no Brasil.” In Psicologia de Família: Teoria, Avaliação e Intervenção, edited by M. L. M. Teodoro and M. N. Baptista , 256–272. Artmed.

[jmft70094-bib-0031] Schmidt, B. , I. M. Silva , A. S. Sehn , M. C. Aires , and A. M. N. Paiva . 2022. “Perda, Luto E Resiliência Na Pandemia De COVID‐19: Implicações Para a Prática Com Famílias.” Pensando Famílias 26, no. 1: 3–14. https://pepsic.bvsalud.org/pdf/penf/v26n1/v26n1a02.pdf.

[jmft70094-bib-0032] Silva, I. M. , S. R. Lordello , and L. Polejack . 2021. Luto na Pandemia Como Intervenção Psicossocial: Uma Proposta Pioneira da UnB. UnB Notícias. https://noticias.unb.br/artigos-main/5083-luto-na-pandemia-como-intervencao-psicossocial-uma-proposta-pioneira-da-unb.

[jmft70094-bib-0033] Terry, G. , N. Hayfield , V. Clarke , and V. Braun . 2017. “Thematic analysis.” In The Sage Handbook of Qualitative Research in Psychology, edited by C. Willig and W. Stainton‐Rogers , 17–37. Sage.

[jmft70094-bib-0034] White, M. 2007. Maps of Narrative Practice. Norton.

[jmft70094-bib-0035] White, M. 2009. Narratives of Therapists' Lives. General Books.

[jmft70094-bib-0036] White, M. 1998. “Saying Hello Again: The Incorporation of the Loss Relationship in the Resolution of Grief.” In Introducing Narrative Therapy: A Collection of Practice Based Writings, edited by C. White and D. Denborough , 17–32. Dulwich Centre Publications.

[jmft70094-bib-0037] White, M. , and D. Epston . 1990. Medios Narrativos Para Fines Terapeuticos. Ed. Paidós.

[jmft70094-bib-0038] World Health Organization . (n. d.). Coronavirus Disease (COVID‐19) Pandemic. https://www.who.int/europe/emergencies/situations/covid-19.

[jmft70094-bib-0039] World Health Organization . 2025. WHO COVID‐19 Dashboard. https://data.who.int/dashboards/covid19/deaths.

